# Automated Peptide Synthesizers and Glycoprotein Synthesis

**DOI:** 10.3389/fchem.2022.896098

**Published:** 2022-05-05

**Authors:** Jiekang Tian, Yaohao Li, Bo Ma, Zhongping Tan, Shiying Shang

**Affiliations:** ^1^ Center of Pharmaceutical Technology, School of Pharmaceutical Sciences, Tsinghua University, Beijing, China; ^2^ State Key Laboratory of Bioactive Substance and Function of Natural Medicines, Institute of Materia Medica, Chinese Academy of Medical Sciences and Peking Union Medical College, Beijing, China

**Keywords:** peptide synthesis, glycoprotein synthesis, automated synthesizer, solid phase peptide synthesis, batch synthesis, continuous flow, high throughput synthesis

## Abstract

The development and application of commercially available automated peptide synthesizers has played an essential role in almost all areas of peptide and protein research. Recent advances in peptide synthesis method and solid-phase chemistry provide new opportunities for optimizing synthetic efficiency of peptide synthesizers. The efforts in this direction have led to the successful preparation of peptides up to more than 150 amino acid residues in length. Such success is particularly useful for addressing the challenges associated with the chemical synthesis of glycoproteins. The purpose of this review is to provide a brief overview of the evolution of peptide synthesizer and glycoprotein synthesis. The discussions in this article include the principles underlying the representative synthesizers, the strengths and weaknesses of different synthesizers in light of their principles, and how to further improve the applicability of peptide synthesizers in glycoprotein synthesis.

## Introduction

**FIGURE 1:**
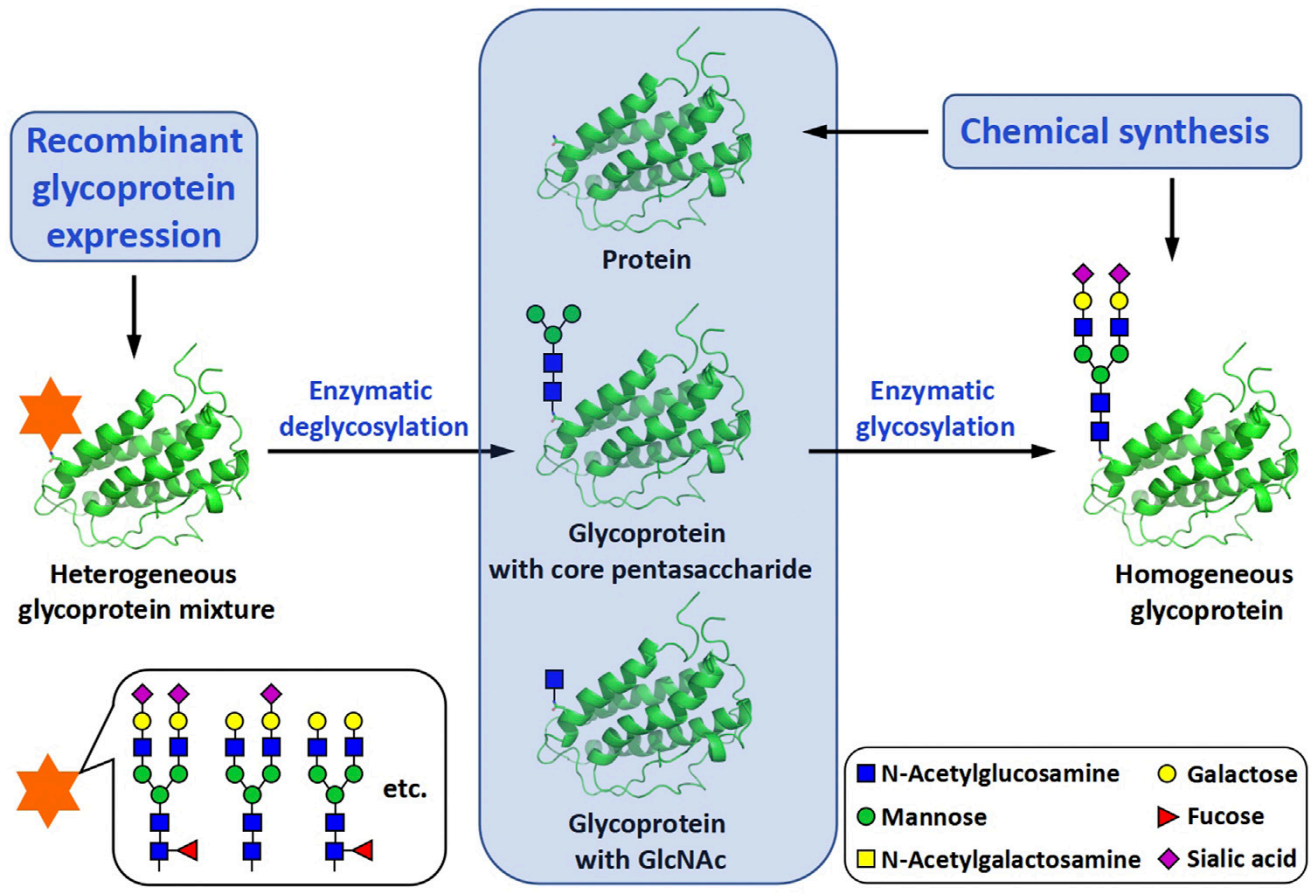
Biochemical and chemical methods for the preparation of homogeneous glycoproteins.

The synthesis of glycoproteins with well-defined protein and carbohydrate structures is essential for the study of their structures, properties and functions (Davis, 2002). To meet with this requirement, many different methods have been investigated for their use in the synthesis of homogeneous glycoforms, *i.e.*, glycoprotein isoforms with the same protein amino acid sequence but different glycosylation patterns. Based on the way of how to incorporate glycans into glycoproteins, these methods can be roughly classified into two categories, biochemical methods and chemical methods (Rich and Withers, 2009). No matter which type of methods is selected, the synthesis is more or less associated with glycopeptide and peptide synthesis (Figure 1).

**FIGURE 2:**
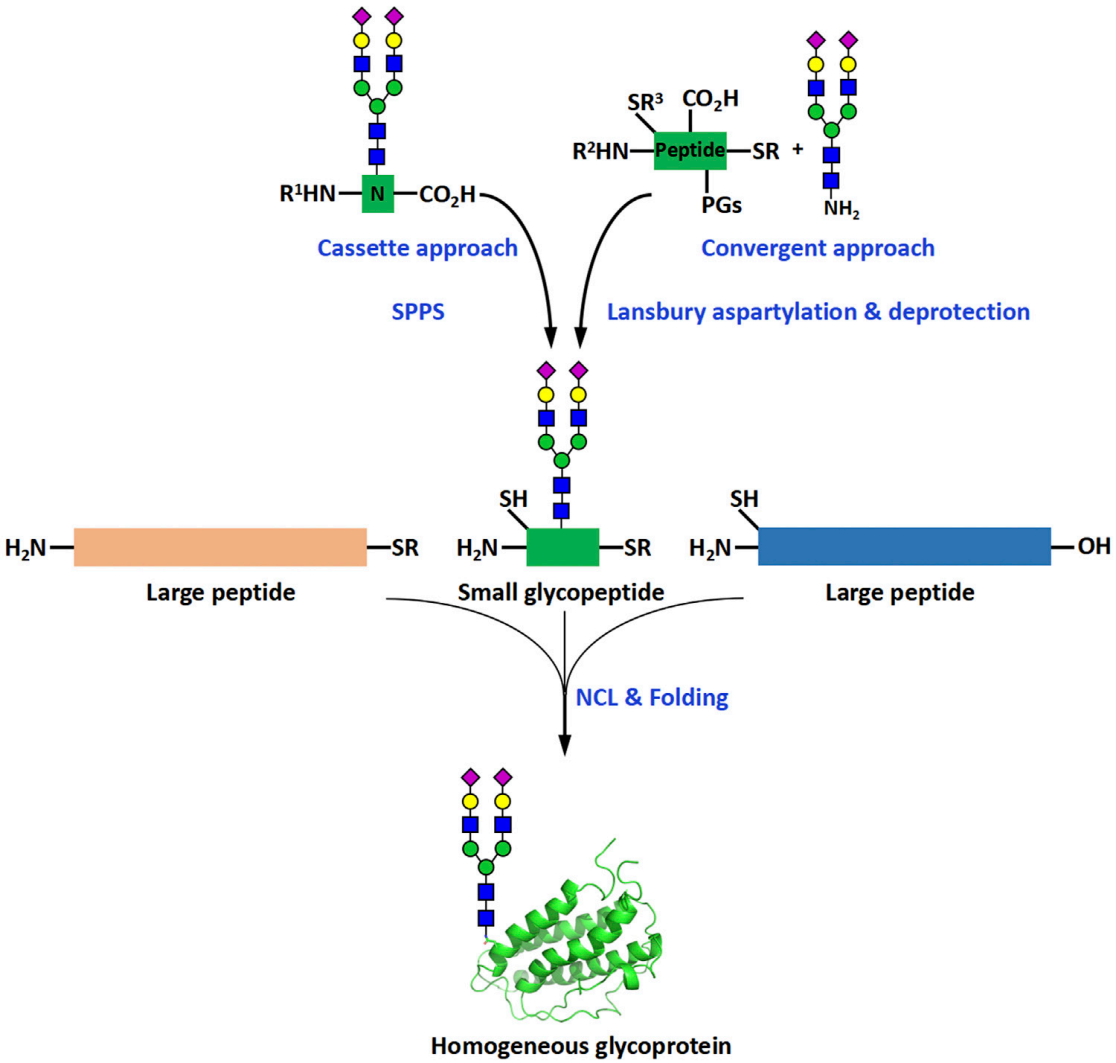
Chemical methods for the synthesis of homogeneous glycoproteins. R, alkyl or aryl groups; R^1^, R^2^, R^3^, protecting groups of amino or thiol groups; PGs, protecting groups of peptide side chains. NCL is the abbreviation of “native chemical ligation”, which is a reaction that is commonly used in assembly of proteins and glycoproteins. It normally involves the chemoselective ligation between a C-terminal thioester and an N-terminal thiol amino acid-containing fragment.

The biochemical methods include two main subtypes, glycoprotein remodeling and enzymatic glycosylation of proteins. Glycoprotein remodeling involves first enzymatic trimming of the heterogenous glycans on a recombinant glycoprotein to produce a homogeneous glycoform or chemical synthesis of a homogeneous glycoform, and subsequent modification of the glycoform containing a core glycan or single N-acetylglucosamine (GlcNAc) residue by the glycosyltransferase- or endoglycosidase-catalyzed stepwise glycan synthesis or one-step transglycosylation (Li and Wang, 2018), while enzymatic glycosylation of proteins uses glycosyltransferases to directly build glycans on recombinant or chemically synthesized proteins (Figure 1). In the chemical methods of glycoprotein synthesis, the desired products are generated by covalently joining the peptide and glycopeptide fragments together using native chemical ligation (NCL) (Dawson et al., 1994) (Figure 2).

Compared with chemical methods, biochemical methods are more convenient to use and more practical in the preparation of large glycoproteins and large-scale preparation of glycoproteins. However, due to the substrate specificity of the enzymatic reactions, the number of glycosylation patterns that can be generated by biochemical methods are limited (Ma et al., 2020). Although tedious and labor-intensive to perform, chemical methods are more flexible and precise, and in theory, can be used to prepare glycoforms with any structures. This advantage is very helpful to gain a more comprehensive and in-depth understanding of the role of protein glycosylation (Chaffey et al., 2018).

Chemical methods typically use NCL reactions to join glycopeptides to peptides to produce homogeneous glycoproteins (Figure 2). NCL reactions are relatively simple to set up and perform, and can be completed quickly, usually in a day. The difficulties associated with the chemical synthesis of glycoproteins are mainly due to two reasons: the high synthetic complexity and the low synthetic efficiency. Most of the complexity comes from the preparation and purification of glycopeptides and the cause of the low efficiency can be attributed largely to the use of relatively short peptides as the synthetic fragments. The use of short peptides increases the number of required synthetic steps and thus leads to decrease in synthetic efficiency. Based on these facts, it is reasonable to expect that small glycopeptide and long peptide fragments would reduce the difficulties in glycoprotein synthesis (Figure 2). Small glycopeptides only contain a small number of amino acids and can be prepared by manual synthesis. Manual synthesis is more likely to make better use of precious synthetic glycans, thereby reducing the time required for repetitive glycan and glycopeptide synthesis. The use of larger peptide fragments, on the other hand, would reduce the number of ligation and purification steps, thereby improving the efficiency of the assembly of glycoproteins. With the continuous improvement of automated peptide synthesizers, the problems associated with the synthesis of large peptides are expected to be slowly addressed. In this review, we provide a brief overview of the development history of peptide synthesizers, with a focus on the progress achieved at each stage of development.

## Early Peptide Synthesizers

Before the 1960s, peptide synthesis was an almost impossible task. In 1954, Vincent Du Vigneaud used the strategy of tetraethyl pyrophosphate-mediated solution-phase coupling reaction and sodium/liquid ammonia deprotection to synthesize oxytocin (Du Vigneaud et al., 1954). It took him many years and much effort to complete the synthesis of this octapeptide hormone. In 1963, Bruce Merrifield turned peptide synthesis from nearly impossible to possible. After more than 4 years of exploration, he developed a new technology called Solid-Phase Peptide Synthesis (SPPS) (Merrifield, 1963). This revolutionary technology allows all steps, including coupling, washing and deprotection, to be carried out in the same reaction vessel without isolation and purification of reaction intermediates, thus greatly simplifying the peptide synthesis process.

**FIGURE 3:**
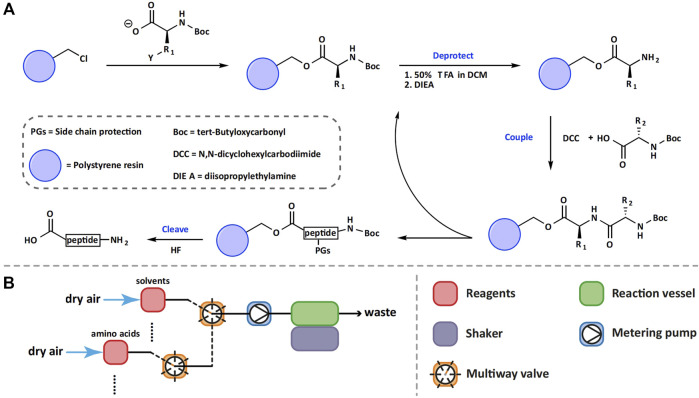
Schematic representation of Boc-SPPS **(A)** and the first automated solid-phase peptide synthesizer **(B)**.

In the process of developing SPPS, Merrifield and his coworkers conducted many optimization studies and established most of the basic principles that still apply today: 1) optimal resins are critical for efficient synthesis of peptides; 2) attaching the C-terminal amino acid to the solid support and extending the peptide chain in the C to N direction to reduce racemization; 3) using orthogonal protecting groups for the α-amines of amino acids to enable the selective formation of amide bonds; 4) increasing the coupling efficiency to be higher than 99%; 5) using strong acids to simultaneously cleave peptides from resins and remove side-chain protecting groups (Figure 3A) (Merrifield, 1965).

In accordance with these principles, Merrifield and coworkers optimized the Boc chemistry for SPPS and established the following conditions as their optimal conditions: SPPS was carried out on a resin obtained by the copolymerization of styrene and divinylbenzene (98% and 2% respectively). Boc was used as the α-amine protecting group, DCC as the coupling reagent, DMF as the solvent, and the solution of HBr or HF in TFA as the cleavage cocktail. Under the aforementioned optimized conditions, SPPS became more routine and easier to perform. In 1964, Merrifield reported the total synthesis of Bradykinin (sequence: RPPGFSPFR), a nonapeptide plasma kinin, using optimized Boc chemistry (Merrifield, 1964). The manual synthesis was carried out on a Boc-L-Arg (NO_2_)-polystyrene-divinylbenzene resin and was completed in 8 days. The yield of the crude peptide was 93% and after purification, the overall yield of the chromatographically pure peptide was 68%. This result clearly demonstrated that the efficiency of solid-phase synthesis of peptides is much higher than that of solution-phase synthesis.

### Automated Peptide Synthesizers Using Boc Chemistry

Although the manual SPPS gave satisfactory results in the synthesis of short peptides, this technology became less practical when applied to the synthesis of large peptides due to the considerable amount of time demanded by the synthesis task. To overcome this issue, in 1965, Merrifield and his collaborators designed and constructed the first automated solid-phase peptide synthesizer based on the use of Boc chemistry (Merrifield and Stewart, 1965). The application of automation to the SPPS process greatly reduced the need for manpower for performing peptide synthesis and human error, and thus significantly improved and simplified the synthesis of long peptides. As shown in Figure 3B, in the automated synthesizer, the resin beads were kept in the same reaction vessel during the entire synthesis process. Metering pumps were used to transfer an appropriate amount of solvent or reagents into the reaction vessel according to the pre-calculated volume and concentration.

Using their newly invented instrument, Merrifield and coworkers did a comparative synthesis of bradykinin. It was found that the synthesis can be completed in 32 h, which is only 20% of the time required in the manual synthesis (Merrifield, 1964). In the following years, they had continuously improved the performance of their synthesizer and was eventually able to produce ribonuclease A, a 124 amino acid-long peptide, in one-shot synthesis (Gutte and Merrifield, 1971).

In the 1980s, the commercialization of the automated peptide synthesizer ABI 430 developed by Applied Biosystems further promoted the synthesis of large peptides. However, as more and more studies were conducted, researchers gradually realized that it is not always possible to synthesize large peptides like ribonuclease A and the length of peptides that could be reliably prepared using automated peptide synthesizers is generally limited to be less than 50 amino acids. This is likely to be related to the batch-wise design of the system, which has the disadvantages of relatively low efficiency of reagent mixing, slow mass transfer and long coupling time (Breen et al., 2021).

### Automated Peptide Synthesizers Using Fmoc Chemistry

During the rapid development of peptide synthesizers based on the Boc chemistry, in 1970, Carpino and Han introduced the Fmoc group for the protection of the α-amines of amino acids (Carpino and Han, 1970). Fmoc can be efficiently removed under mild basic conditions like 20% piperidine in DMF. When Fmoc is chosen for the protection of the amine function of the amino acid, the TFA-labile groups like Boc, Trt, and Pbf can be used to protect the side chains, thus avoiding the need for the highly corrosive and toxic acid HF in the final cleavage step of SPPS. In addition, the fluorescence property of the dibenzofulvene adducts formed after treatment of the Fmoc resin with piperidine enables the estimation of the efficiency of each peptide coupling step.

In the early 1970s, a method for thoroughly mixing resin beads with solvent and reagents, the continuous flow SPPS method, was developed by Bayer et al. (1970) *.* Unlike the batch-wise SPPS method adopted by the peptide synthesizers like ABI 430, which maintains the reaction suspension by gas bubbling or mechanical stirring, the continuous flow method uses a pump to provides a rapid and continuous flow through the reaction vessel, thus increasing the efficiency of mixing and mass transfer, and decreasing the coupling time (Lukas et al., 1981).

Using Fmoc chemistry and continuous flow technology, PerSeptive Biosystems developed the Pioneer Peptide Synthesis System, an automated synthesizer capable of performing the peptide synthesis in a simple and straightforward manner. This instrument uses glass column with filters at the top and bottom as the reaction vessel. Pumps and valves are used to control and regulate the flow of solvent and reagent solutions. A distinguishing feature of this peptide synthesizer is that it has a UV detector. The coupling efficiency of each step of SPPS can be assessed based on the intensity of the UV signal, which in turn can direct the optimization process to look for solutions to improve the quality of peptide products.

Slightly different from the methods used for Boc SPPS, pseudoproline dipeptides, Dmb/Hmb-protected dipeptides and isoacyl dipeptides are often required for peptide synthesis using Fmoc chemistry (Behrendt et al., 2016). This is mainly due to the different aggregation behaviors of the growing peptide chains on resin in the presence or absence of TFA. During Boc SPPS, the TFA-protonated N-terminal amino group can generate electrostatic repulsion between peptides and consequently expose the reactive groups for the following coupling reactions. During Fmoc SPPS, the lack of electrostatic repulsion can increase the risk of aggregation and reduce the synthesis efficiency. The use of dipeptides can alleviate this issue by inhibiting aggregation of peptides. However, because most peptides do not contain enough sites that are suitable for the incorporation of dipeptides, the length of peptides that can be successfully synthesized by the Pioneer Peptide Synthesizer is also limited to less than 50 amino acids (most commonly 30–40 residues in length).

### High-Throughput Automated Peptide Synthesizers

Many studies such as mimotope screening require the simultaneous preparation of a large number, but not large quantities of peptides. When tens to hundreds of peptides are needed, it becomes impractical to use 1-channel (ABI 430) or 2-channel (Pioneer) peptide synthesizers for their synthesis. To meet with the high demand in the peptide research community and pharmaceutical companies, new technologies like parallel and combinatorial synthesis of peptide libraries were developed. Among these technologies, the “tea-bag” and the split-and-pool methods have received much attention.

The “tea-bag” method for parallel synthesis of a large number of peptides was developed by Houghten and co-workers (Pinilla et al., 1996). In this method, the sealed polypropylene mesh packets are filled with resin beads and are labeled. Depending on the sequences of the peptides, the packets are placed in different reaction vessels for coupling with the desired amino acids. In this way, each packet can be made to contain only one peptide, whose identity can be determined by the label.

The solid-phase split-and-pool combinatorial peptide synthesis method was reported by Furka et al. (1991). In this method, the resin beads are split into several portions, each reacting with a different amino acid. After the coupling reaction, all the beads are pooled together and resplit into a new set of subgroups to react with different amino acids. As this cycle repeats, the number of the generated peptides increases exponentially. Using the split-and-pool method, it is possible to synthesize millions of peptides in a relatively short period of time, however, the identity of each peptide is unknown.

The complexity of the “tea-bag” and the split-and-pool methods makes it almost impossible to develop automated peptide synthesizers to realize these concepts. Most of the high-throughput peptide synthesizers were designed based on simple parallel synthesis methods. For example, the Advanced ChemTech APEX 396 Automated Multipeptide Synthesizer is capable of the parallel synthesis of 96 peptides. The delivery of reagents and solvents to 96 reaction vessels is accomplished using pipetting robotic arms. The waste generated in the reaction and washing steps is removed from the bottom of the reaction vessels by pressure from the top. Upon completion of the synthesis, the resin needs to be transferred from the reaction vessel of the automated synthesizer to another container for cleavage. Although this type of synthesizers can be well applied in the synthesis of large number of short peptides, they are generally not suitable for the preparation of large peptides.

## Application of Automated Peptide Synthesizers in Glycoprotein Synthesis

Fmoc chemistry was more widely used in the synthesis of glycoproteins. The most important reason behind this preference is the fact that glycans are not stable under strongly acidic conditions. If Boc chemistry is used, the repetitive TFA acidolysis employed for the Boc-group deprotection and HF employed for final cleavage can cause deleterious side reactions to complex glycans on glycopeptides. In addition, the reaction conditions in Fmoc SPPS are much milder than those in Boc SPPS and the liquid waste generated in Fmoc SPPS is environmentally more friendly. Therefore, Fmoc chemistry is generally used for the synthesis of glycoproteins.

In the process of previous glycoprotein chemical synthesis, large peptide fragments were generally directly prepared using automated peptide synthesizers. The synthesis of glycopeptides was more complicated. Depending on the types of glycans and how glycans were made, the methods for glycopeptide synthesis can be divided into two categories. The most common method for preparing O-linked glycopeptides was to directly use Fmoc-protected glycosylated amino acids as building blocks for automated SPPS (Figure 2). This “cassette approach” was also applied to N-linked glycoamino acids that are isolated from natural sources like chicken eggs (Seko et al., 1997; Sun et al., 2014; Liu et al., 2017). The preparation of most N-linked glycans requires time- and labor-intensive chemical synthesis. The glycopeptides bearing the chemically synthesized glycans were mostly synthesized by convergent approaches like the Lansbury aspartylation reaction, which involves the direct coupling of a glycosylamine with the side-chain carboxyl group of the Asp residue in the peptide (Figure 2). After the synthesis of the large peptide fragments and glycopeptide fragments, they were joined together by NCL to afford the desired glycoprotein products (Li et al., 2017).

### O-Linked Glycoprotein Synthesis

In the past 20 years, a few O-linked glycoproteins have been prepared by chemical synthesis (Marcaurelle et al., 2001; Asahina et al., 2019; Wang et al., 2021; Zhao et al., 2022) and a representative one is lymphotactin (Lptn), a 93-amino acid chemokine that contains eight O-glycosylation sites at its C-terminus. In 2001, Bertozzi and coworkers reported its synthesis for the first time (Marcaurelle et al., 2001).

**FIGURE 4:**
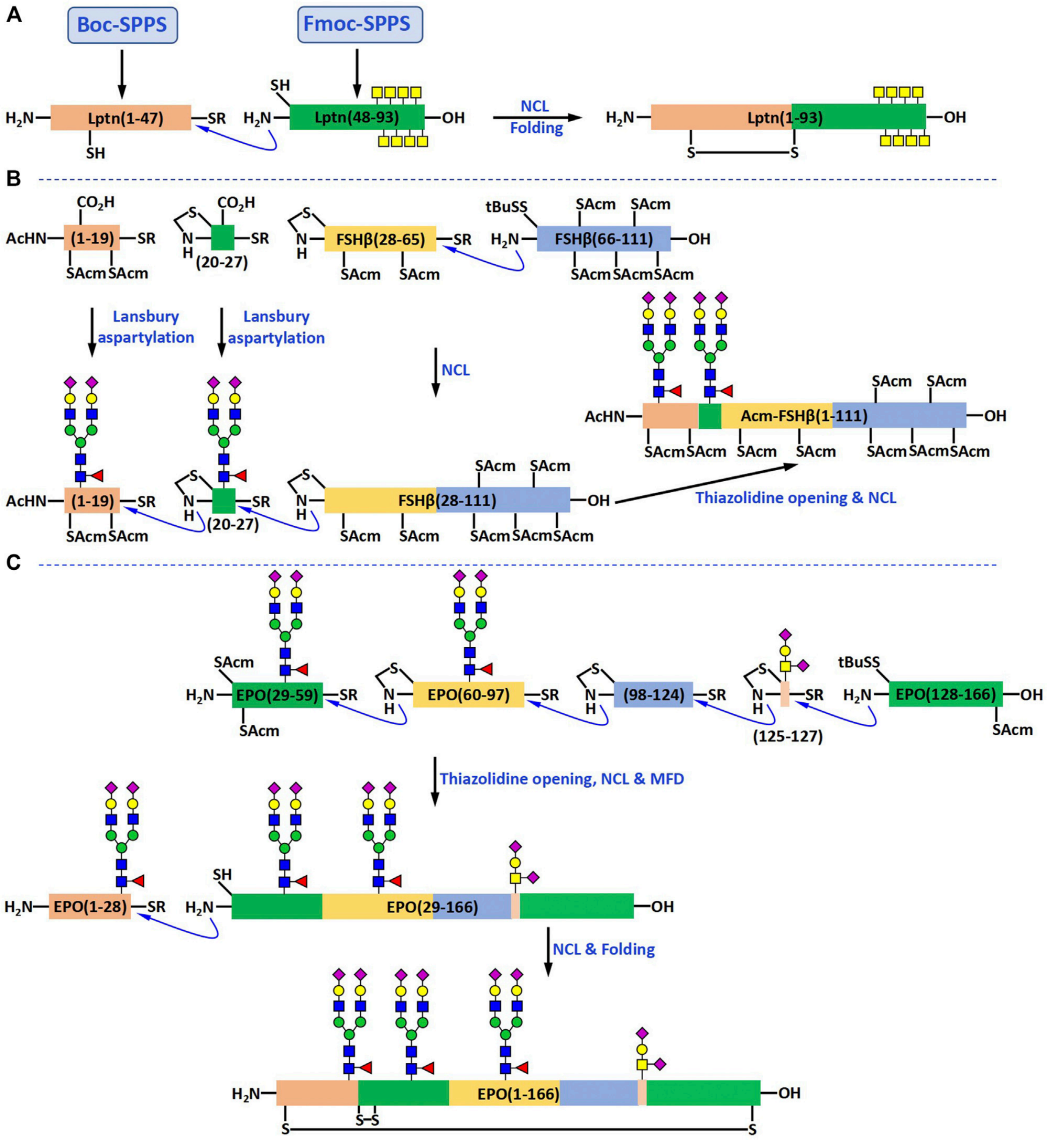
Chemical syntheses of Lptn bearing O-glycans **(A)**, FSHβ bearing N-glycans **(B)** and EPO bearing both O- and N-glycans **(C)**.

According to the retrosynthesis strategy based on NCL, Lptn can be generated by combining two fragments: one fragment is a 47-amino acid long peptide Lptn (1-47), and the other fragment is a 46-amino acid long glycopeptide Lptn (48-93), which contains eight O-linked GalNAc residues. They first attempted to prepare Lptn (1-47) with a C-terminal thioester using Fmoc SPPS (Figure 4A). However, their experimental results showed that the desired product could not be detected. To solve this problem, they turned to the Boc SPPS, which enabled them to obtain the peptide thioester directly.

The glycopeptide fragment Lptn (48-93) was prepared using the cassette approach mentioned above. Fmoc-Gly Wang resin and Fmoc chemistry were used in the synthesis. In order to achieve a reasonable coupling efficiency of the sterically hindered glycosylated amino acid building blocks at the glycosylation sites Thr 76, Thr 79, Thr 81, Ser 84, Thr 85, Thr 87, Thr 90, and Thr 92, manual synthesis was carried out for the first 20 amino acids. HBTU and HOBt were used as coupling agents, and the Kaiser test was used to verify the completion of each coupling step. After the synthesis of the first half of the glycopeptide, the resin beads were transferred to the reaction vessel of an ABI 431A automated peptide synthesizer to complete the second half. The coupling agents used on the peptide synthesizer was DCC and HOBt. The glycopeptide fragment was cleaved from the resin using Reagent K and the acetyl groups on GalNAc residues were removed with the aqueous solution of hydrazine hydrate (10%). RP-HPLC purification afforded the glycopeptide product Lptn (48-93) in 24% yield.

The NCL of the large peptide Lptn (1-47) with the glycopeptide Lptn (48-93) was carried out by mixing these two fragments at a ratio of approximately 1:1 in the ligation buffer. They were slowly ligated together to form Lptn (1-93) as the major product, which was isolated and purified by RP-HPLC in 38% yield. The successful folding of Lptn (1-93) was realized by dissolving this large glycopeptide in a cysteine/cystine redox buffer. After another RP-HPLC purification, the desired glycoprotein product was obtained in 49% yield (Figure 4A).

### N-Linked Glycoprotein Synthesis

Many N-linked glycoproteins have also been synthesized in the past decade (Piontek et al., 2009a; Piontek et al., 2009b; Izumi et al., 2012; Sakamoto et al., 2012; Okamoto et al., 2014; Reif et al., 2014; Wang et al., 2020; Li et al., 2021). The synthetic strategies for the preparation of most of them are very similar to that used for generating O-glycosylated Lptn, with the only difference being that the building blocks become the N-glycoamino acids isolated from chicken egg yolk powder or soybean powder (Izumi et al., 2012; Sakamoto et al., 2012). The number of N-glycopeptides that were prepared by the direct use of the Lansbury aspartylation reaction is relatively smaller than that by the “cassette approach”. In 2012, Danishefsky and coworkers reported the synthesis of the β-subunit of follicle-stimulating hormone (FSHβ) in which the Lansbury aspartylation reaction was employed for the preparation of the N-glycopeptide fragments (Nagorny et al., 2012). FSHβ consists of 111 amino acids and two N-linked glycans at residues Asn7 and Asn24. According to their retrosynthetic analysis, FSHβ could be obtained from four fragments, two glycopeptide fragments FSHβ (1-19) and FSHβ (20-27), and two peptide fragments FSHβ (28-65) and FSH β (66-111) (Figure 4B).

The first step in the synthesis of glycopeptide FSHβ (1-19) was the SPPS of Fmoc-FSHβ (1-18). The Fmoc-Arg (Pbf)-TGT resin and the Pioneer Peptide Synthesizer were employed for the synthesis of this peptide. After Fmoc SPPS and resin cleavage by acetic acid, the Phe phenylthioester was attached to the C-terminus of the protected-peptide under the condition optimized by Sakakibara et al. Convergent coupling of the synthetic N-glycan with the peptide thioester *via* the Lansbury aspartylation reaction gave the desired glycopeptide product in 17% yield. The glycopeptide FSHβ (20-27) bearing the same N-linked dodecasaccharide was prepared in a similar way and obtained in 27% yield. The two peptide fragments FSHβ (28-65) and FSHβ (66-111) were synthesized using the Fmoc-Gly-TGT resin and Fmoc-Glu (OtBu)-TGT, respectively. The overall yield for FSHβ (28-65) was 48% and for FSHβ (66-111) 31%.

Under NCL conditions, the two peptide fragments FSHβ (28-65) and FSHβ (66-111) were first ligated together to form the large peptide FSHβ (28-111) in 38% yield. Using a similar ligation approach, the glycopeptide fragment FSHβ (20-27) was added to the N-terminus of FSHβ (28-111) in 26% yield, and the glycopeptide FSHβ (1-19) to the N-terminus of FSHβ (20-111) in 27% yield. The resulting large glycopeptide FSHβ (1-111) was not folded to generate the final glycoprotein product.

### Synthesis of Glycoproteins Containing Both O- and N-Linked Glycans

Many natural glycoproteins contain both O- and N-glycans. However, due to its difficulty, there are only a few reports on the synthesis of these glycoproteins in previous studies (Wang et al., 2013; Fernandez-Tejada et al., 2014; Ye et al., 2021). In 2013, Danishefsky and coworkers completed the synthesis of a glycoprotein with such complexity, human erythropoietin (EPO) (Wang et al., 2013). EPO has 166 amino acids and four glycosylation sites, three N-glycosylation sites at Asn24, Asn38, and Asn83, and one O-glycosylation site at Ser126. Four glycopeptide fragments EPO (1-28), EPO (29-59), EPO (60-97), and EPO (125-166), and one peptide fragment EPO (98-124) were used for the synthesis of this glycoprotein (Figure 4C). The peptide fragments were prepared *via* Fmoc SPPS using a Pioneer Peptide Synthesizer. The N-glycopeptides were generated by coupling the synthetic dodecasaccharide anomeric amine with different peptide thioesters by the Lansbury aspartylation reaction. The O-glycopeptide was obtained by ligating the glycosylated EPO (125-127) with EPO (128-166).

After the synthesis of all five fragments, four of them were joined together in the C to N direction using NCL to generate the glycosylated EPO (29-166). The four non-native Cys residues in this glycopeptide, which were introduced to facilitate NCL reactions, were quantitatively desulfurized to Ala by the metal-free desulfurization (MFD) reaction. The Acm groups on the native Cys residues were then removed exposing the N-terminal reactive site, which underwent another NCL reaction to generate the large glycopeptide EPO (1-166). The folding was carried out in a cysteine/cystine redox buffer and the correctly folded glycoprotein product was purified by RP-HPLC.

## Peptide Synthesizers With Heating

Most of the peptide synthesizers used in the reported synthesis of glycoproteins were developed more than 20 years ago. Although the synthesis could be achieved using these instruments, the synthetic efficiency is not high enough and the synthetic yields for peptides longer than 30 amino acids were generally lower than 50%. Such low yields led to overall low efficiency in glycoprotein synthesis. In order to improve the synthesis of glycoproteins, as we suggested above, it is necessary to not only significantly optimize the methods for the synthesis of glycans and glycopeptides (*i.e.*, development of more efficient manual synthetic methods), but also the methods for the synthesis of large peptides.

In recent years, many different methods have been explored to further improve the performance of automated peptide synthesizers in the preparation of large peptides. One simple and reliable approach is to heat the reactions in SPPS. The effect of an elevated temperature during SPPS has been controversial since it was originally proposed. On one hand, peptides are complex and may undergo side reactions at high temperature. On the other hand, as the length of the peptide chain increases, the intra- and intermolecular aggregation can significantly reduces the reaction efficiency, and increasing temperature may be a solution to this issue.

The microwave-assisted peptide synthesis has attracted much attention in the past decade. At the beginning of the development of this technology, it has been speculated that there are so-called non-thermal microwave effects on the synthesis because of the observed acceleration in rate and alteration in product distribution as compared with conventional heating (De La Hoz et al., 2005). However, more and more experiments indicate that there is no essential difference between different heating methods. The observed changes may be the results of thermal/kinetic effects (Bacsa et al., 2008).

**FIGURE 5:**
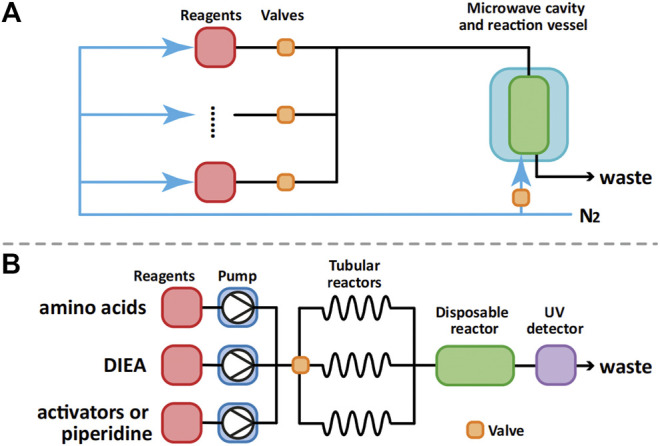
Process flow diagram of CEM Liberty **(A)** and AFPS developed by Pentelute et al **(B)**.

Currently, the two major manufacturers of fully automated microwave-assisted peptide synthesizers are CEM and Biotage. Possibly due to the limitations of current microwave technology, the more efficient continuous flow method was not used in their synthesizers. Instead, they used nitrogen bubbling (CEM Liberty, Figure 5A) and vortexing (Biotage Syro Wave) to mix resin beads with solvent and reagents during SPPS. Another crucial limitation of microwave-assisted peptide synthesizers is their inability to monitor the progress of the synthesis visually.

Compared with the batch mode synthesis used in the microwave-assisted peptide synthesizers, the flow mode synthesis is more efficient in heat transfer, more economical and more flexible (Plutschack et al., 2017). Recently, Pentelute et al. demonstrated the advantages of flow peptide synthesis. Using their automated fast-flow synthesizer (AFPS, Figure 5B), they were able to successfully prepare a series of long peptides such as sortase A (59–206), which is 164 amino acids long (Hartrampf et al., 2020). Although the requirement of the use of large excess of amino acid building blocks (>40 eq) in the synthesis limits the application of such a peptide synthesizer in the synthesis of glycopeptides, its advantage in the preparation of large peptides is expected to have a positive impact on glycoprotein chemical synthesis.

## Conclusion and Perspectives

Although several decades of efforts and development have led to great achievements in glycoprotein synthesis, many challenges still exist in this research area. Currently, the chemical synthesis of glycoproteins is a complex, expensive and time-consuming process. In order to address this issue, optimization of the automated peptide synthesis is required. A possible solution is to apply the newly developed automated fast-flow peptide synthesizers to perform the synthesis of large peptide fragments. It is expected that if such synthesis is reliably realized, it should greatly promote the advancement of glycoprotein synthesis in the future.
